# Telemedicine supported by Augmented Reality: an interactive guide for untrained people in performing an ECG test

**DOI:** 10.1186/1475-925X-13-153

**Published:** 2014-11-21

**Authors:** Paolo Bifulco, Fabio Narducci, Raffaele Vertucci, Pasquale Ambruosi, Mario Cesarelli, Maria Romano

**Affiliations:** Department of Electrical Engineering and Information Technology, University of Naples “Federico II”, Naples, Italy; VRLab, University of Salerno, Salerno, Italy; Selex ES, Giugliano, Italy

**Keywords:** Augmented Reality, Untrained user, ECG device operation, Electrode placement

## Abstract

**Background:**

In many telemedicine applications, the correct use of medical device at the point of need is essential to provide an appropriate service. Some applications may require untrained people to interact with medical devices and patients: care delivery in transportation, military actions, home care and telemedicine training.

Appropriate operation of medical device and correct connection with patient’s body are crucial. In these scenarios, tailored applications of Augmented Reality can offer a valid support by guiding untrained people at the point of need. This study aims to explore the feasibility of using Augmented Reality in telemedicine applications, by facilitating an acceptable use of biomedical equipment by any unskilled person. In particular, a prototype system was built in order to estimate how untrained users, with limited or no knowledge, can effectively interact with an ECG device and properly placing ECG electrodes on patient’s chest.

**Methods:**

An Augmented Reality application was built to support untrained users in performing an ECG test. Simple markers attached to the ECG device and onto patient’s thorax allow camera calibration. Once objects and their pose in the space are recognized, the video of the current scene is enriched, in real-time, with additional pointers, text boxes and audio that help the untrained operator to perform the appropriate sequence of operations. All the buttons, switches, ports of the ECG device together with the location of precordial leads were coded and indicated. Some user’s voice commands were also included to improve usability.

**Results:**

Ten untrained volunteers, supported by the augmented reality, were able to carry out a complete ECG test first on a mannequin and then on a real patient in a reasonable time (about 8 minutes on average). Average positioning errors of precordial electrodes resulted less than 3 mm for the mannequin and less than 7 mm for the real patient. These preliminary findings suggest the effectiveness of the developed application and the validity of clinical ECG recordings.

**Conclusion:**

This application can be adapted to support the use of other medical equipment as well as other telemedicine tasks and it could be performed with a Tablet or a Smartphone.

## Background

Telemedicine refers to the use of telecommunications and information technologies for the delivery of medical services where is needed [1–3]. For many applications, the correct usage of medical device at the point of need is essential to provide an appropriate service, but there are some practical situations that may require untrained or inexpert people to interact with medical devices and patients. Some examples are telemedicine services on transportation (e.g. aircrafts [4–6], boats [7, 8], trains, etc.), application during military actions [9], on islands or remote areas [10, 11], some emergency applications [12, 13], but also home care telemedicine supported by family members [14–16], elderly care [17, 18], operators training and so on.

In these cases, untrained or improvised (but necessary) actors can, involuntarily, use medical instruments in an inappropriate manner and/or make improper connection between the patient and the medical device seriously invalidating the telemedicine service.

In these scenarios, tailored applications of Augmented Reality (AR) can offer a valid support by guiding non-trained people to a correct usage of medical devices at the point of need. Augmented reality basically consists of a live view of the real-world in which some elements of the scene are “augmented” (enriched, enhanced) by computer-generated information such as graphics, texts and sounds. The application domains for AR are numerous and extend in different fields such as training and support, design, medicine, entertainment and cultural heritage [19–21].

Recently, there is a growing interest about AR in medicine. The main applications of AR are in the field of surgery, rehabilitation and teaching/training. Interventional medicine, surgery [22, 23], laparoscopy and other procedures (e.g. needle biopsy [24]) can be assisted by integrating preoperative and intraoperative anatomic and functional data improving the visual perception of the surgeon [20, 25–27]. Obviously, surgery AR applications require very accurate registration and camera calibration [28]. AR and virtual reality have long since found use in rehabilitation and particularly in neurorehabilitation, by guiding and aiding the patient to perform therapy [29, 30]. Teaching and training of students or physicians can take great advantage by AR, which can be further enriched with direct haptic and auditory feedback [31–33]. Superimposition in real time of images from US, CT or MR scans can also help in learning [20].

There are only very few examples of AR applications in telemedicine, among these there are systems of virtual reality supporting distance teaching of minimally invasive surgery and systems for interactive telemedicine in the operating theatre [34, 35]; some low-cost peripherals to support telehealth, visualization, education and collaborative systems [36]; some applications of distance training for the restoration of motor function, supported by virtual reality [37].

The capability of AR to provide live support to users in operating on instrumentations is also of interest for this study. AR can support and guide workers in operating, actuate, disassembling and maintaining complex devices or systems. As example, it was proposed a mixed reality environment aimed to improve the effectiveness of servicing and repair procedures in mission critical systems, while reducing the time required for the intervention. Also technicians with no previous experience on specific, complex devices were able to perform an assigned maintenance task when supported by the AR application. In maintenance activities there are well-defined sequences of procedures to be done in a relatively static environment. These features allow a defined design space, supporting a wide variety of systems and technologies. It was proved effective in supporting workers for maintenance activities [38].

As feasibility study for new possible telemedicine services, an AR application was built to support untrained users in performing an ECG-test. In particular, the objective of this study was to analytical assess the benefits of exploiting augmented reality principles in order to estimate how untrained users with limited or no knowledge can properly interact with an ECG device and properly placing ECG electrodes on patient’s chest. The proposed AR application was assessed in terms of efficacy and clinical acceptability.

In many clinical activities, 12-lead electrocardiogram is an essential medical investigation. On the other hand, it should carefully carried out. Misplaced ECG electrodes can cause changes in ECG recordings, which could have an impact on clinical decisions [39]. Incorrect electrode cable connections, reversal of electrodes, inadequate placement of the electrodes are common source of error (changes the true ECG morphology) and can conceal or simulate different pathology such as, myocardial ischemia or infarction, arrhythmias, ventricular hypertrophy [40–43]. It is also worth mentioning that untrained service providers are one of the key barriers to implementation of telemedicine services.

## Methods

### Overall system description

**Figure 1:**
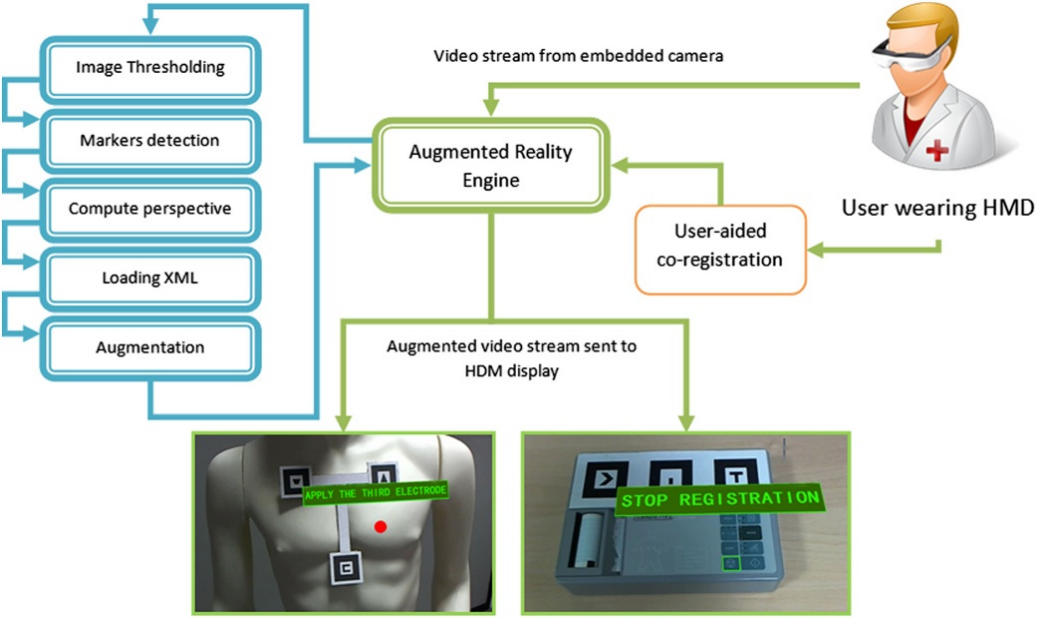
**System architecture.** Scheme of the operations of the developed AR application to guide an untrained user to correctly perform an ECG test.

The developed AR application guides the user to perform a predefined sequence of simple tasks on the ECG device (e.g. connect cables, press buttons, check indicator lights, etc.) and on the patient (e.g. connect electrodes in a specific position, etc.), driving user’s attention from time to time to the relevant item.A flow-chart of activities was created to describe all possible sequences of simple tasks necessary to carry out an electrocardiographic test using that particular ECG device. The developed AR application drives the user to perform each predefined task by presenting text, graphics and audio messages to him/her (see Figure 1).

Specifically designed sets of markers were attached to the ECG device and to the patient: this allows the AR engine to evaluate on real-time the 3D pose of these objects with respect to the user.This permits to indicate or highlights a specific point or a part of the objects (e.g. a button to be pressed) by superimposing opportune signs to the current scene. The operators worn a Head Mounted Display (HMD) coupled to a webcam in order to see the virtual contents that augment the current scene (future developments will involve the use of a single Tablet or Smartphone). Figure 1 presents a general scheme of the proposed AR application.

### The augmented reality engine

The Augmented Reality engine is in charge of user’s head tracking and scene augmentation/rendering. The tracking system of the developed application is based on the ARToolKit, an open source AR library [44]. The ARToolKit video tracking libraries compute the current camera position and orientation relative to physical markers in real time. Very simple markers (i.e. black squares containing specific identification symbols) are used. Markers are rigidly attached to the objects of interest to estimate user’s perspective, i.e. the relative 3D pose of these objects with respect to the camera (camera calibration). For each captured frame, the AR engine performs a defined sequence of steps. First of all, it looks for known markers in the framed scene. A thresholding operation turns the coloured image into a binary one in order to make the detection of the markers easier and faster. Once detected all visible markers, the engine computes the user’s perspective view (i.e. user’s relative 3D positioning with respect to the marked objects). At this stage, the AR engine loads pre-recorded information about each marked object (e.g. its geometry, the internal spatial coordinates of its parts, etc.) and then is possible to superimpose virtual objects (e.g. labels, arrows, spots etc) to the real scene. The “augmented” scene is finally displayed to the user by means of proper displays, such as the HMD.

In normal environmental conditions (i.e. acceptable lighting of the scene, absence of strong reflections or excessive shadows, etc.), a single marker could be enough to achieve a reliable augmentation of the scene. However, the marker should be always in the central field of view of the camera and should be entirely and clearly visible to reduce the risk of detection miss. In several contexts, arranging a marker in the middle of operational field could simply be unfeasible or it could even interfere with the operations. Furthermore, object characterized by uneven surfaces or significant rotations of displacements can hinder a continuous and stable detection of the user’s perspective.

Previous studies [45] have shown that the use of multiple markers (or a constellation of markers) for each object of interest can address most of the problems arising in practical situations, thus delivering an inherently more robust and more accurate tracking of object, even by using small markers. A useful advantage of the multi marker approach is that it is easily scalable. In fact, by adding other markers it is possible to easily widen the tracking volume as desired. The AR application includes a calibration function meant to measure and compensate the geometrical distortion generated by the lens of the camera. In addition, a specific procedure allows a manual fine tuning and coregistration between the camera and its virtual counterpart in charge of rendering the required graphics. Each of the six degrees of freedom, including the focal length of the camera and also the threshold used for marker identification, can be precisely adjusted. This task is performed only once, generally, on the very first system start-up. An ad-hoc graphic user interface supports this fine tuning calibration procedure. This procedure can be recalled when the equipment configuration is changed. The AR application memorizes and exchanges information using XML (eXtensible Markup Language) files. The language is widely used and it allows extensibility, unambiguously and large compatibility. All information of the accurate location of any relevant element of the objects and of the working environment is memorized in a database of XML files. More technically, an XML object file defines the object’s points of interest. These include Cartesian coordinates and properties of augmented aids, such as the location of textual information, audio messages, and the interaction region on the object surface to be highlighted. The AR engine builds up the virtual scene by means of a DOM (Document Object Model) XML parser. To find the required data in the application database, a XML-based XPath query language is used. Combining the information from tracker and from XML database, the AR engine is therefore able to locate user’s perspective view in the real world and to extract all the required augmented contents from the repository to properly guide him/her during the intervention.

### AR application development and set-up

The AR application has to support an untrained user to perform an ECG-test on a patient. Starting from the established medical guidelines and technical instructions of the device exploited, each ECG procedure was carefully analyzed and subdivided in very simple tasks (or steps), achieving the whole formalization of the all possible sequences of steps. Once we obtained the whole flow chart, it was coded as a deterministic finite automaton (DFA) that has a finite number of possible states and precise rules for stepping form one state to another, producing unique runs of the automaton. Each procedure is an automaton run that starts at the root and ends in a leaf. A particular state represents a precise ECG-test step and the links with other steps define the order of the execution of the entire procedure. DFA results particularly suited to model either simple or complex procedures in an easy and comprehensive way. The DFA representation was also translated into several XML files where each one represents a run of the automaton or, on the other hand, a possible procedure. The syntax of this file is very simple, a tag < step > defines an elementary task of any procedure. Once the parser reaches the end of the XML document, the procedure is completed.

User is invited to execute each task by an audio message, while a correspondent text-box is presented on the user’s screen. Simultaneously, real scene is augmented by adding graphics (i.e. pointers, spots, etc.) to drive user’s attention on a particular point of the objects (e.g. the button to be pressed, etc.). Once accomplished the task, user sends a vocal command to the AR application to step to the next task. Only very few vocal commands are allowed such as: “Go Next” to pass to the next step; “Go Back” to return to the previous step; “Redo Instruction” to listen to once again the voice prompt of the current step.

Basically, the ECG-test procedure includes: connection of the electrodes to the patient (including the six precordial leads V1-V6), connection of the patient cable to the ECG device, a sequence of operation on the ECG device leading to record 12-leads ECG on a strip chart.

**Figure 2:**
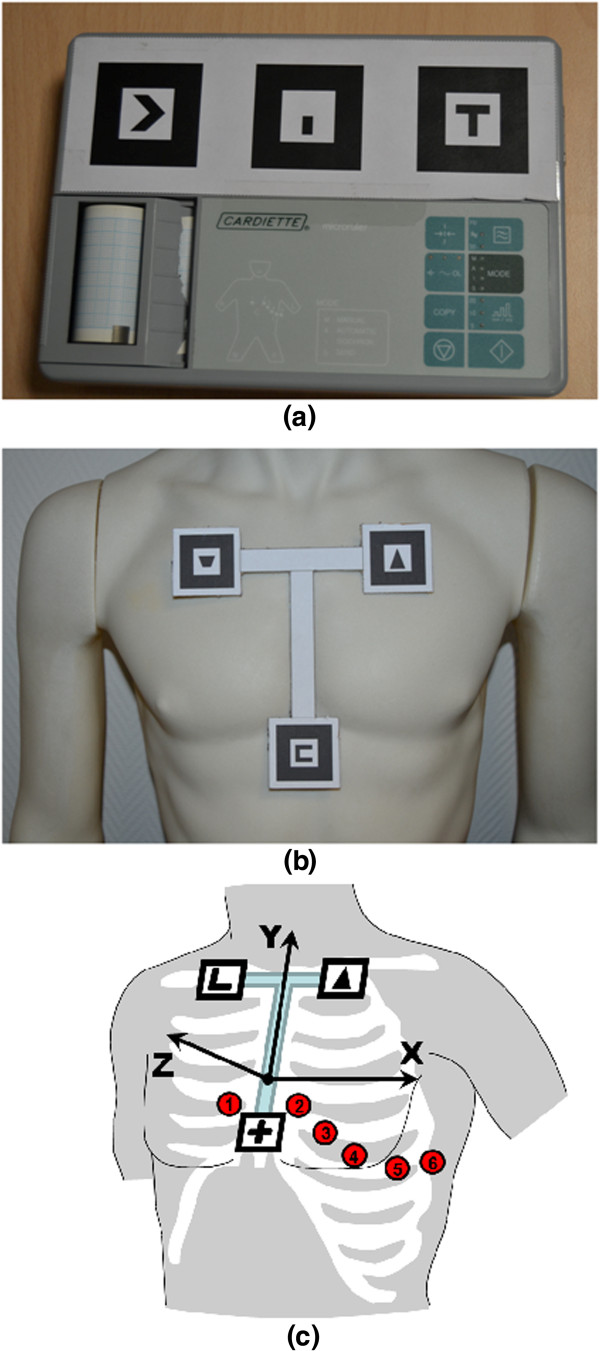
**Portable ECG with markers.** The portable ECG device adopted for this study with attached on the correspondent three markers. The side of each marker measures 4 cm **(a)**. The rigid T-shaped frame enclosing the three markers placed on the thorax of a mannequin. The upper side of the lowest marker is placed in correspond to the xiphoid process **(b)**. Schematic representation of the reference frame adopted in the trials and the relative positions of the precordial electrodes V1, V2, V3, V4, V5 and V6 **(c)**.

Each marker used to augment an object is a black square (of 4 cm per side) containing a unique identification symbol. Markers can be easily printed on paper or cardboard and have to be attached on the object (markers should be rigid and fixed with respect to the object to be augmented). To allow more reliable and stable functioning, a costellation of three markers was associated to the ECG device and a different set of three to the patient’s thorax. The multi-markers approach significantly improves the accuracy of the augmentation [46] compared to single marker approaches [47, 48], thus reducing the matching error between the rendering of the augmenting point and its physical location in the real scene. The portable ECG device considered in this application was a Cardiette Microruler 12/1. The three markers were arranged in line and horizontally aligned, spaced from each other by 2 cm and attached to the top of the front part of the ECG device (see Figure 2a). The geometrical localization of the following parts of the ECG device was accurately measured with respect to central point of the markers costellation (located in the geometrical center of the middle marker) and then recorded in XML file: the main switch (placed on the left lateral side of the device), the connector of the patient cable (placed on the right lateral side), all the buttons (eight in total, placed on the main panel), all the LEDs (twelve in total, placed on the main panel), the accessible parts of the chart printer (placed on the main panel).

**Figure 3:**
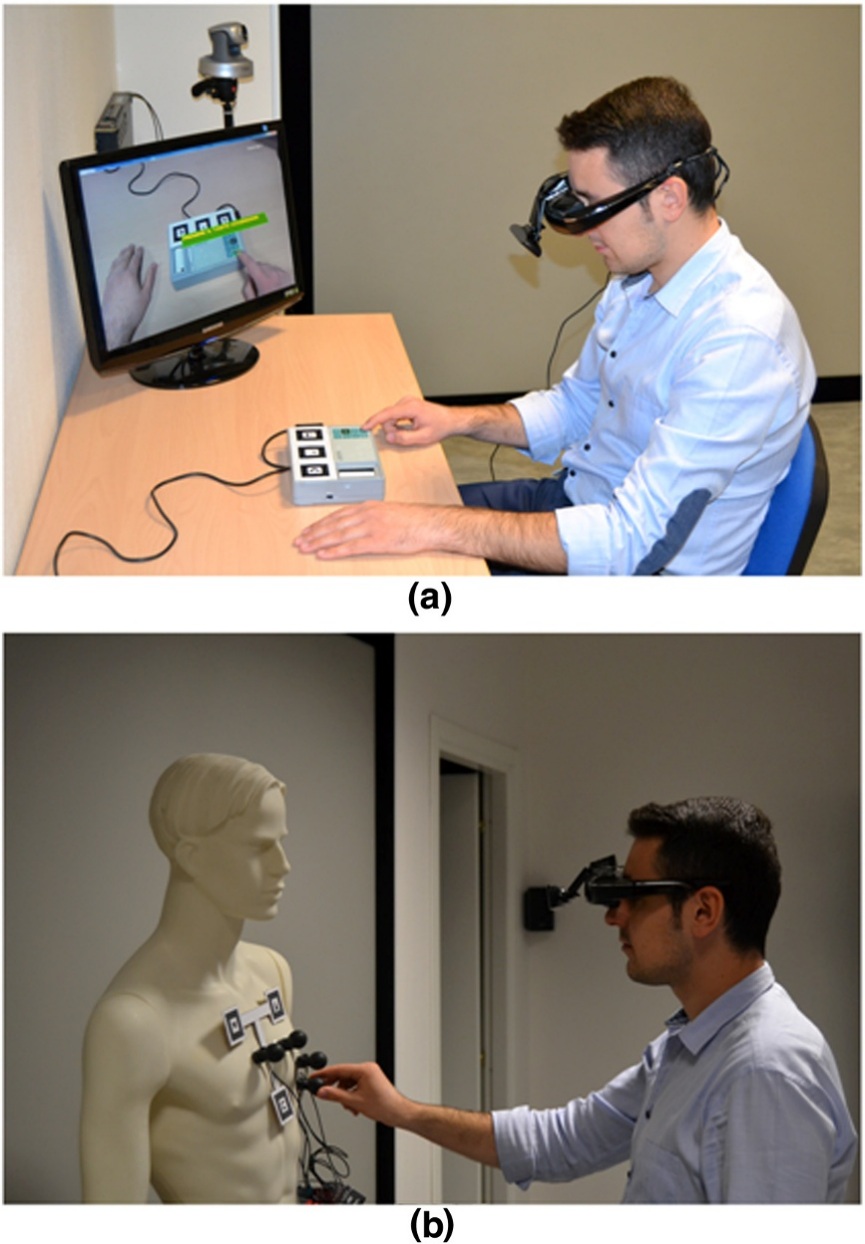
**Untrained user on a mannequin and on the portable ECG.** Pictures representing an untrained user while placing precordial electrodes on the mannequin **(a)** and operating the ECG-device **(b)**.

For the augmentation of the patient’s thorax, the three markers were arranged on the tips of a T-shaped stucture showed in Figure 2b. The T-structure was made flat and rigid, its horizontal and vertical segment of the T-structure measured 10 cm (empirically chosen according the mean size of adult’s thorax [49]). The T-structure was applied with stickers onto the patient’s thorax, taking care to align the vertical segment along the sternum and positioning the upper side of the lower marker (recognizable in figure by its C-like symbol) in correspondence of the xiphoid process (the lower end of the sternum), which can be easily recognizable by touching (see Figure 2b). The anatomical landmarks for the standard precordial electrode positions were fixed in accordance with the current ECG international standards [50]. The precordial electrode positions are: V1 and V2 at the fourth intercostals space to the right and left sternal border, respectively; V4 at the fifth left intercostal space in the mid-clavicular line; V3 midway between V2 and V4; and V5 and V6 at the horizontal level of V4 in the anterior and midaxillary lines, respectively [39, 51]. Coordinates x, y and z were obtained for each of the precordial electrodes with respect to the reference system fixed to the T-shape marker set (see Figure 2c) and opportunely coded in the XML file. The x-, y- and z-axis correspond to the latero-lateral, cranial-caudal and dorsal-ventral patient’s anatomical axes, respectively.The user (see Figure 3a and 3b) worn an HMD (Silicon Micro Display ST1080) offering a 1920x1080 pixels video resolution at 60Hz, 45 degrees diagonal field of view, 24 bit stereo audio and a weight of 180 grams. To provide a vision in augmented reality through the HMD, it was coupled with a Logitech HD Pro Webcam C910 that offers a very wide diagonal field of view of 83 degrees, 4.3 mm focal length and a maximum video resolution of 1920x1080 pixels. However, in order to get the best results in term of augmentation of the scene, we preferred to use a 640x480 pixels video resolution that allows to work at 30 FPS.

### Experiments setting

**Figure 4:**
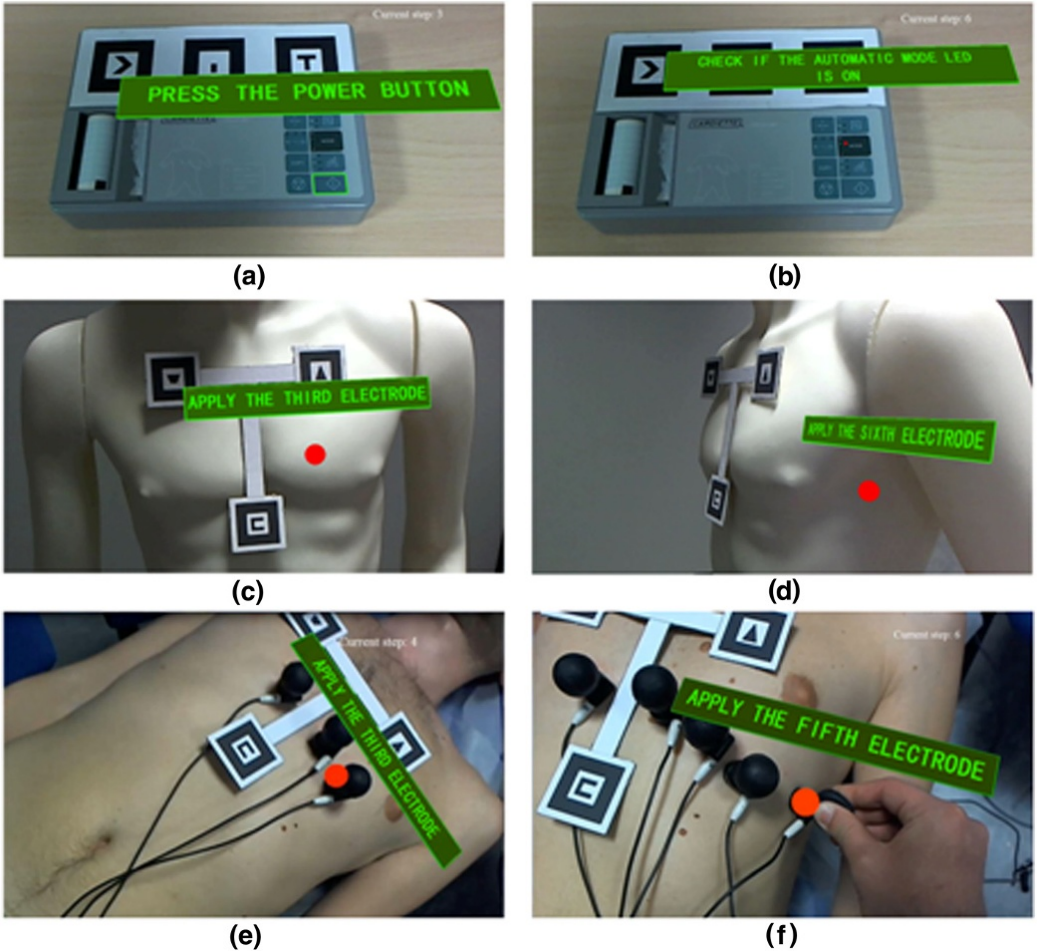
**Examples of augmented scenes.** Examples of augmented real scenes: the power button of the ECG device is surrounded by a light green rectangle, while audio and text invite the user to press it **(a)**; a little red spot indicates the LED to be checked by the users **(b)**; the location of the V3 precordial electrode on the mannequin is highlighted with a red circle **(c)**; the location of the V6 precordial electrode is highlighted with a red circle **(d)** please note the inclination with respect to the plane of the markers; the location of the V3 precordial electrode on a real patient is highlighted with a red circle **(e)**; placement of the V5 precordial electrode on the patient’s thorax, the location of V5 is highlighted with a red circle **(f)**.

To assess the feasibility of the AR application and its benefit, two separated experimental tests were designed: the first on a life-size mannequin and the second on a real patient. The experimental tests were carried out involving two groups of 10 people each (14 men and 6 women in total, mean age of 31.3 and median age of 30.5) with no medical expertise, no experience of ECG test and who never used an ECG device. At each tester was asked to wear the HMD and, after familiarizing with the system for few minutes, the experimental trial was started. The first group of 10 people carried out complete ECG recording sessions interacting with the ECG device and placing electrodes on the mannequin (see Figure 4a-d).The second test was performed on a volunteer, male adult acting as patient who lay supine on a table and breathed normally to resemble a practical case (see Figure 4a-b, 4e-f). Similarly, the second group of 10 untrained people was asked to perform complete ECG recordings only supported by the AR application.

The ability to perform the required operations, the time required to complete the ECG recording, the positioning errors of the electrodes and the user’s judgment were collected for all the tester. In particular, the three spatial components of the distance between an expected (true) location of an electrode and its actual placement were recorded as the error committed by the untrained user. This information was used to quantitatively assess the efficacy and the clinical acceptability of the developed AR application.

## Results and discussion

Each of the untrained tester was able to carry out in an appropriate manner the ECG-test with the only support of the AR application. All the testers reported that they had not encountered any particular difficulty in interacting with the AR application and in carrying out all the requested actions. Some of them reported only minor problems in perception of distances in the direction perpendicular to their plane of view. This is due to the use of a single camera, which obviously fails to accurately render the depth of the scene. The AR application was perceived as intuitive and easy to use, the opportunity to interact with vocal commands was particularly appreciated.

**Figure 5:**
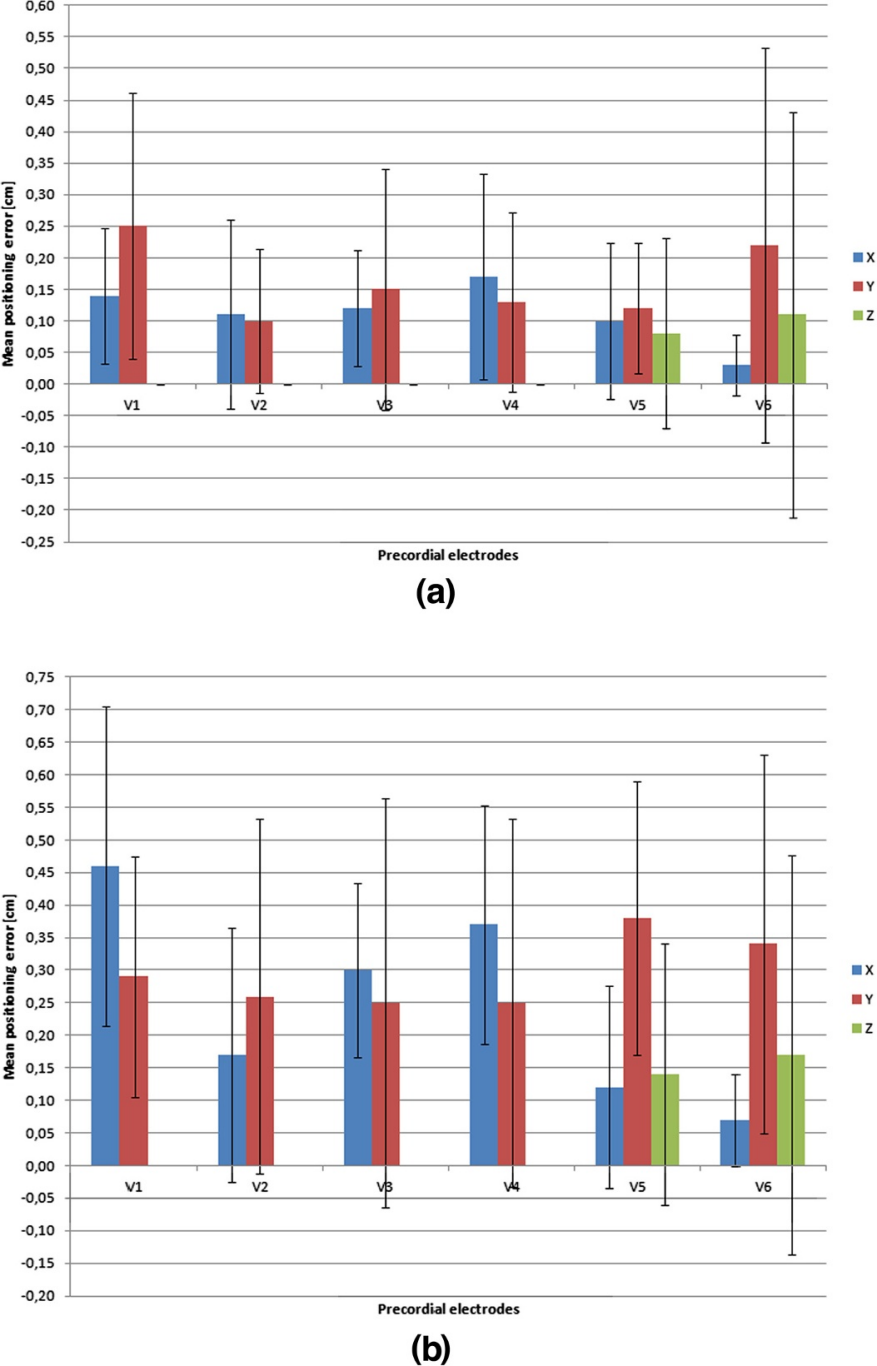
**Average x, y and z components of positioning errors for precordial electrodes.** Averages (bars) and Standard Deviations (segments) of the errors (distance from the expected location reported in centimeters for each of the x, y and z axes of the space) in the placement of the precordial electrodes on the mannequin **(a)** and on the patient’s chest **(b)** by the ten untrained users. V1, V2, V3, V4, V5 and V6 are the six precordial locations;

The execution time over both tests was on average of 8 minutes (including 3 minutes of ECG recording in the automatic mode) that can be considered acceptable for practical purposes.Bar plots in Figure 5 show the averages and standard deviations of the x, y and z components of errors (computed on all testers) in placement of the precordial electrodes on the mannequin (Figure 5a) and patient’s chest (Figure 5b). Averages can be considered as an indication of the accuracy, while standard deviations as a measure of the precision (repeatability) achieved during the trials.

In addition and in a more concise way, Table 1 reports errors measured as distance between the expected and the occurred position for each of the precordial electrodes V1-V6. The average, standard deviation and maximum of these displacement errors are available for both tests (mannequin and real patient).

**Table 1:** **Positioning errors for precordial electrodes**

		Precordial lead					
Test		V1	V2	V3	V4	V5	V6
**Mannequin**	**Average error [cm]**	0.30	0.18	0.23	0.24	0.22	0.27
	***± SD of the error [cm]***	*± 0.10*	*± 0.10*	*± 0.13*	*± 0.19*	*± 0.11*	*± 0.41*
	**Max error [cm]**	0.45	0.40	0.45	0.54	0.41	1.42
**Patient**	**Average error [cm]**	0.40	0.27	0.44	0.46	0.49	0.69
	***± SD of the error [cm]***	*± 0.07*	*± 0.20*	*± 0.20*	*± 0.22*	*± 0.18*	*± 0.28*
	**Max error [cm]**	0.50	0.73	0.78	0.81	0.79	1.56

Average, standard deviation and maximum of the displacement errors done by the ten users of the developed AR application in placing precordial electrodes (V1-V6) during the tests on mannequin and real patient. All data are expressed in centimeters. The displacement errors were computed as the square root of the sum of the square of the three Cartesian components (x, y, and z) of the displacement vector.

For the first test, the average errors in electrode positioning on the mannequin resulted less than or equal to 3 mm, while the standard deviation less than 5 mm.

Involvement of the real patient instead of the mannequin led to a slight increase of the errors committed. This can be reasonably due to thorax motion due to breathing or other little movements of the patient. Nevertheless, even in the test of the real patient the mispositioning of the electrodes resulted less than 7 mm on average and reached a maximum of 16 mm on V6, these data support the effectiveness of the AR procedure and the clinical acceptability of the recorded ECG. As a matter of fact, electrode malposition exceeding 25 mm is associated with potentially significant ECG changes [39]. Taking into account this threshold value and observing the results achieved during both tests (Figure 5a-b and Table 1), the average errors in electrode positioning resulted reasonably acceptable and comparable with placement errors usually made by technicians and nurses in an emergency care department [52]. This supports the clinical validity of the acquired ECG waveforms by means of the developed AR system.

It is worth noting that in both tests only for V5 and V6 significant variations of positioning were registered on the z-axis. This is inherent to the specific positions of these two electrodes and their relative positioning with respect to the markers on the thorax. When the user directs his gaze towards V5 or V6, the plane on which lies the marker (x- and y-axes represented in Figure 2c) results significantly angled and then the 3D pose errors increase [48]. This mainly occurs because of the natural curvature of the human thorax. Indeed, many studies have shown that the accuracy of positioning virtual object on the real scene mainly depends on camera distance and viewing angle with respect to the markers and also on other factors (e.g. size of the marker, focal length, field of view, pixel resolution, etc.) [48, 53, 54].

However, in the specific case, the distance and the relative angle between camera and markers are actually limited. Indeed, when placing the electrodes onto a patient or when manually operating on the electrocardiograph, the distance between the camera and markers cannot be greater than the length of operator’s arm, nor the angle of gaze be particularly tilted. Hence, the need for manual interaction with objects marked severely restricts the space in which augmented reality operates.

Due to the perspective of the scene taken by the camera, the greatest pose error of an AR marker lies in the direction that connects the marker with the camera. However, in this specific application (e.g. in placing the electrodes V1 to V4) the error made on the z-axis of Figure 2c (coincident with the most probable line of sight of user) results in practice negligible for the precordial electrodes V1 to V4: the user’s hand must stops on patient’s skin. This, obviously, does not hold for the electrodes V5 and V6 because of their intrinsic positions. Consequently, the placement of these electrodes suffers from greater errors (an higher component on z axis, see Figure 5a-b, and consequently an increase in the positioning error, see Table 1).

Lastly, it is also interesting to note that the errors are consistent with data reported by the developers of the system ARToolKit [48]. Indeed, if we consider that usually the operator is located at approximately 30-40 cm from the patient (and therefore from the markers) with an angle between 0° and 45° the errors predicted by previous studies [48, 53] are contained within +/- 5 mm; while for a greater angle (e.g. 45°-80°) they increase to approximately +/-12 mm. In the extreme case in which the inclination of the user’s gaze line reaches or exceeds 90 degrees with respect to the marker plane, the markers are no longer in sight making impossible to coherently augment the scene. Even if this event did not occur during the trials, it is possible and should be taken into account.

## Conclusion

This study has highlighted the possibility of using Augmented Reality to support untrained user while performing an ECG-test. The developed AR application is at the proof-of-concept stage and can certainly be improved. Furthermore, it can be easily generalized to the use of other medical equipment.

AR can have a relevant and positive impact on various telemedicine applications. For example, telemedicine applications involving acquisition of diagnostic signals onboard of a plane or a ship by an untrained crew member or even passengers, as well as in the battlefield by untrained soldiers where it can obtain very beneficial effect. Also other home-care solutions, where a relative may be request to interact with medical device or emergency application (e.g. use of a defibrillator by an inexpert user), can take advantage of such AR applications. More appropriate and reliable usage of medical device can be obtained, but also opens new horizons for novel and/or more advanced telemedicine applications.

**Figure 6:**
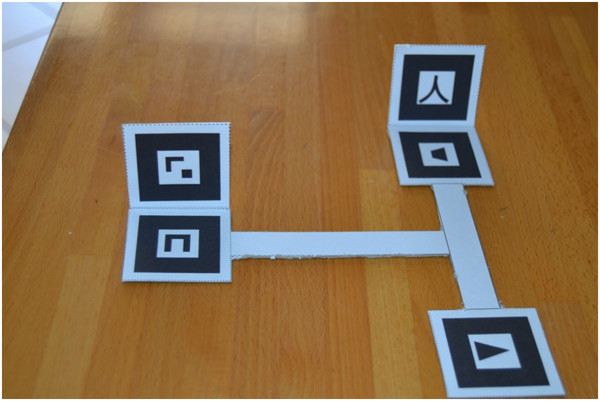
**An improved marker set.** Picture of the improved T-structure including the two extra markers oriented perpendicularly to the patient’s coronal plane.

**Figure 7:**
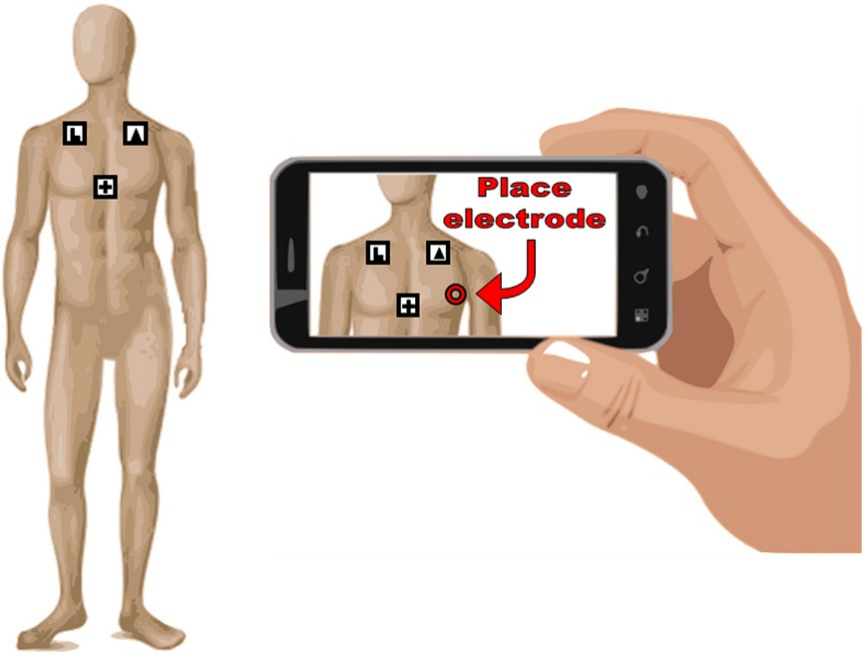
**Possible AR application on smartphones.** Possible, future scenario involving the use of the proposed AR application on smartphones.

However, some issues and question should be fully addressed before moving to clinical applications. While no particular difficulties arose in using AR with medical device (mainly because they are rigid and their 3D geometry known and fixed), problems occur when “augmenting” a generic, alive, human body: large anatomical variations and patient’s motion can be accurately taken into account. It is worth to remember that even if some parts of the human body can be roughly considered rigid, change in proportions should be accounted, e.g., between adults of infants, but also between different body types. Some parts of the body are in continuous motion such as the chest during breathing. However, in the conducted trials, with the patient breathing normally, this phenomenon did not significantly affect the results. These considerations lead to the use of non-rigid constellations of markers (e.g. proportionally scalable in geometric proportions) to take into account different body types, or even to a marker-less recognition of the body (however, this requires the use of sophisticated software and can adversely affect the real-time operation). The final AR application should also consider and properly instruct the operator on how to face with special situation (e.g. in the case of very hairy patients). The flexibility of the XML language offers the possibility to easily enrich or to customize the set of operations and warnings. For example, a simple reminder for the operator to shave the skin or clip small patches of hair for proper sticking of electrodes, whether it is the case, can be included in the final AR application.This study, being essentially a proof of concept, does not provide solutions to these problems. Only a small variation in the markers T-structure has been designed to overcome the problem of excessive user’s slant. An improved version was obtained by adding two extra markers placed adjacently to the left marker (that indicated by the trapezoidal symbol) and the lower marker (that indicated by the C-like symbol), but arranged at 90 degree with respect to the T-structure plane (i.e. laying in anatomical sagittal planes). Figure 6 shows the new marker constellation for the patient’s body. Very preliminary results seem to confirm the expectations.Further developments of this study are oriented towards the realization of this AR application on mobile devices, i.e. tablets or smartphones (see Figure 7). These devices intrinsically incorporate a camera, a relatively large screen, a processor capable of running the software, and other multimedia interfaces such as speakers and microphone. Possibly, the software can be also downloaded, at the very moment, from the Internet. The use of tablets or smartphones, today widely disseminated, could bring further benefits.

## Consent

Written informed consent was obtained from the patient for the publication of this report and any accompanying images.
